# The Insulin Receptor: An Important Target for the Development of Novel Medicines and Pesticides

**DOI:** 10.3390/ijms23147793

**Published:** 2022-07-14

**Authors:** Xiaohong Zhang, Xuezhen Zhu, Xiaoyang Bi, Jiguang Huang, Lijuan Zhou

**Affiliations:** Key Laboratory of Natural Pesticides & Chemical Biology, Ministry of Education, South China Agricultural University, Guangzhou 510642, China; zhangxh1cau@gmail.com (X.Z.); xuezhenzhuscau@gmail.com (X.Z.); bixyscau@gmail.com (X.B.)

**Keywords:** insulin receptor, function, agonists, antagonists, mechanism, medicine, pesticide

## Abstract

The insulin receptor (IR) is a transmembrane protein that is activated by ligands in insulin signaling pathways. The IR has been considered as a novel therapeutic target for clinical intervention, considering the overexpression of its protein and A-isoform in multiple cancers, Alzheimer’s disease, and Type 2 diabetes mellitus in humans. Meanwhile, it may also serve as a potential target in pest management due to its multiple physiological influences in insects. In this review, we provide an overview of the structural and molecular biology of the IR, functions of IRs in humans and insects, physiological and nonpeptide small molecule modulators of the IR, and the regulating mechanisms of the IR. Xenobiotic compounds and the corresponding insecticidal chemicals functioning on the IR are also discussed. This review is expected to provide useful information for a better understanding of human IR-related diseases, as well as to facilitate the development of novel small-molecule activators and inhibitors of the IR for use as medicines or pesticides.

## 1. Introduction

The insulin receptor (IR) is a transmembrane protein and part of the tyrosine kinase receptors (RTK). It exists as covalently bound receptor dimers at the cell surface [1]. The IR plays essential roles in metabolism, cell growth, and development by transmitting the binding of extracellular ligands into several intracellular signaling cascades [2,3,4]. Previous studies have demonstrated that ligands and the insulin signaling IR are highly conserved among human beings and insects [5,6,7].

In human beings, the function of the IR has been studied for many years, and it has been found to play a crucial role in multiple chronic diseases, including Alzheimer’s disease (AD) [8], Type 2 diabetes mellitus (T2DM) [9,10], and various cancers [2,11,12,13], as well as neurodegenerative disorders [14] and metabolic syndromes [15]. For T2DM, the destruction and dysfunction of pancreatic β-cells are common occurrences, and insulin injection is the only choice for glycemic control [16]. The dramatic increase in T2DM over the globe has led to increasing requirements for insulin. Moreover, insulin injection may require more than one shot each day, is hazardous and inconvenient, causes tissue irritation, abscesses, discomfort, etc., and local allergic reactions, lipoatrophy, lipohypertrophy, etc., are common complications of subcutaneous injections [17,18]. Because of the multiple problems associated with insulin injection, orally active insulin-mimetic compounds would be an ideal substitute [19]. For cancer, IR makes an attractive anticancer target owing to its overexpression in a variety of cancers, especially prostate and breast cancers [20]. Therefore, regulators of the IR, such as β-site amyloid precursor protein cleaving enzyme 1 (BACE1), have been regarded as potential therapeutic target [20,21]. Similarly, IR modulators such as ceritinib and anti-idiotypic antibody AK98 (an off-target IR inhibitor) have been suggested as promising drugs for the treatment of brain tumors and breast cancer, respectively [22,23].

In insects, current evidence points to the roles of the IR in regulating development, reproduction, lifespan, caste differentiation, and wing polyphenism [24,25,26]. To our knowledge, neonicotinoid insecticides (e.g., imidacloprid) are selective agonists of the nicotinic acetylcholine receptor (nAChR) that have been widely used to control various insects [27]. Likewise, ryanodine and diamides are commercial insecticides that are antagonists or activators of insect ryanodine receptors (RyRs) [28]. It may be deduced that modulators of the IR that selectively activate or inhibit the IR may be of considerable value in providing promising drugs for the control of human disease, or as insecticides for the control of insects [29]. In this regard, medicines specifically targeting the IR are diverse. However, IR-targeting insecticides are still lacking. Owing to the persistent use of traditional synthetic insecticides, insect resistance has become increasingly serious. Therefore, there is a growing need for new insecticides with new mechanisms of action. Thus IR-targeting insecticides represent an opportunity in the research and development of insecticides.

In this review, we summarize recent studies focused on the structure, conformation, and modulators of the IR in order to provide insight into the mechanisms of its activation and regulation, which is indispensable for understanding the functions of the IR in human and insect physiological processes, including development, differentiation, metabolism, and aging. This information may facilitate better understanding of IR regulation, ligand specificity, crosstalk, and signaling of IR homologues. We also aim to present the available information that may be useful for the discovery of novel medicines and insecticides.

## 2. Biology Studies of the IR

### 2.1. Molecular Structure of the IR

Biochemically, the IR is encoded by a single gene. The coding region of the IR gene has 22 exons and 21 introns [30]. The alternative splicing of exon 11 encodes a 12-amino-acid sequence at the C-terminus of the α-subunit of the IR gene during transcription, resulting in the formation of the isoforms IR-A and IR-B [31]. IR-B is a mature isoform due to the fact that it includes the 12-amino-acid sequence, while the fetal isoform IR-A does not [10,32]. Both isoforms are expressed in most of the cells associated with energy homeostasis, such as adipocytes, hepatocytes, myocytes, and placenta vascular endothelium; however, they present different functional features [10,33]. Several in vitro and in vivo studies have confirmed that the expression and response of the two isoforms are different in breast cancer and T2DM [11]. IR-B possesses important metabolic functions and is the dominant isoform [2]. Conversely, the less-differentiated isoform IR-A is principally expressed in cancer cells [32]. Activation of IR-A promotes the growth of the cancer cells [34].

**Figure 1:**
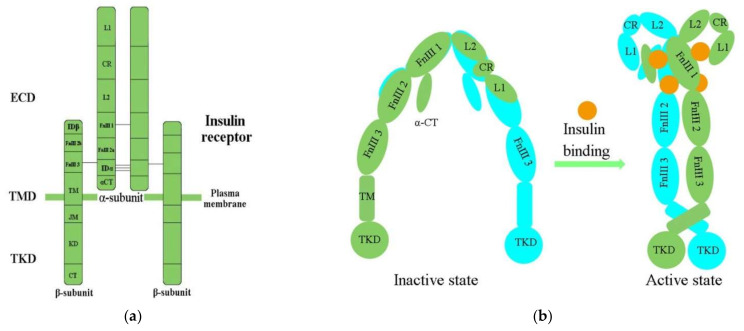
(**a**) The architectural domain of the IR (αβ) _2_ homodimer. Black lines indicate the intersubunit disulfide bonds; (**b**) Inactive and active states of the IR; L1, L2, leucine-rich repeat domains 1, 2; CR, cysteine-rich domain; FnIII-1, 2, 3, fibronectin type-III domains 1, 2, 3; αCT, α C-terminal regions; TM, transmembrane; JM, juxtamembrane; KD, kinase domain; CT, C-terminal tail; ECD, ectodomain; TMD, transmembrane domain; TKD, tyrosine kinase domain.

IR structural studies have previously been described in detail [35,36,37,38] (Table 1). The IR is a glycosylated, disulfide-linked (αβ)_2_ transmembrane homodimer consisting of two repeated ectodomains (ECD), a single transmembrane helix, and two intracellular cytoplasmic domain that includes a tyrosine kinase domain (TKD) (Figure 1a) [38,39]. The α-subunit constitutes most of the IR-ECD, while the β-subunit is necessary for the IR-ECD, the transmembrane domain (TMD), and the intracellular TKD [38].

Determination of the three-dimensional (3D) crystal structure of the insulin-free IR-ECD through crystallography has revealed that the IR-ECD dimer roughly displays an inverted “U”- or “V”-shaped architecture [37,40]. Specifically, L1 and CR together with L2 form one leg, while the linearly arranged FnIII domains form the other leg [37]. However, the modular organization of the ECD, with high intrinsic flexibility and its complex ligand-binding properties, poses a challenge for structural studies of the IR. Furthermore, single-particle cryo-electron microscopy (cryo-EM) has revealed that the IR-ECD dimer converts the overall architecture from an autoinhibited inverted “V” shape into a “T”-shaped conformation, which was stabilized after binding insulin molecules to the N-terminal domains (Figure 1b) [39,41,42]. The L1, CR, and L2 domains of both IR promoters constitute the “T” horizontal part, while the FnIII-1, -2, and -3 domains of the IR dimer constitute the vertical piece of the “T” [42].

Previous biochemical and mutagenesis models of insulin binding have identified two distinct binding sites on both the IR and insulin, termed site 1 (S1) and site 2 (S2) [36]. The L1 subdomain and the α-CT helix residue have been confirmed to represent IR S1 site (IR-S1) [39,45,46]. Evidence has indicated that IR-S1 is indispensable for insulin binding, and minor modifications of it were sufficient to change the IR’s specificity for insulin [47]. Scapin (2018) has defined the full S2 binding site [39], and Gutmann (2020) first observed the connection of insulin with discrete IR-S2 [43]. Studies have demonstrated that optimal IR activation requires multiple insulin molecules bound to S1 and S2 [48,49]. A similar result has also been presented in a study of the cryo-EM structure of the IR–insulin complex at 3.2 Å resolution [42]. The binding of insulin to S1 of apo-IR could release the autoinhibited conformation, which was an essential step for IR activation, while binding to S2 was important for the IR to adopt the active T-shape [50,51,52]. Cryo-EM analysis of the insulin–IR complex has revealed that insulin binds independently to the site of S2 between the FnIII-1 and FnIII-2 domains [43]. Another study has shown that the fibronectin domain is folded inwards, in a pincer-like fashion, which brings domains FnIII-3 and FnIII-3′ into contact [44].

The cryo-EM structure of the full-length human IR–insulin complex (human HEK293F cells) in the active state at an overall resolution of 3.2 Å unexpectedly revealed that a maximum of four insulin molecules can bind to the “T”-shaped IR dimer at four distinct sites [42]. Furthermore, at least one insulin molecule bound to two S2s and a maximum of four insulin molecules at four sites are required to form the “T”-shaped dimer [39,42,52]. Insulin 1 mainly binds to the primary site formed by the L1 domain, and α-CT then makes contact with a loop of the FnIII-1 domain from the IR promoter that donates α-CT [42]. During IR activation, a tripartite interface between insulin 1 and site 1 stabilizes the active IR dimer. Insulin 2 binds to a novel binding site on the FnIII-1 domain, located on the backside of the β sheet [42].

However, there is still a lack of detailed analysis of which site is connected first and how the first and second insulin binding results in different phosphorylation status of the IR [52]. The reported findings have emphasized the importance of the conformational changes of the IR-ECD and IR–insulin complex in the insulin/insulin-like growth factor signaling (IIS) pathway. Hence, the precise mechanism of how insulin binds to the IR at first remains elusive, and further research is still needed.

### 2.2. Activation of the IR 

**Figure 2:**
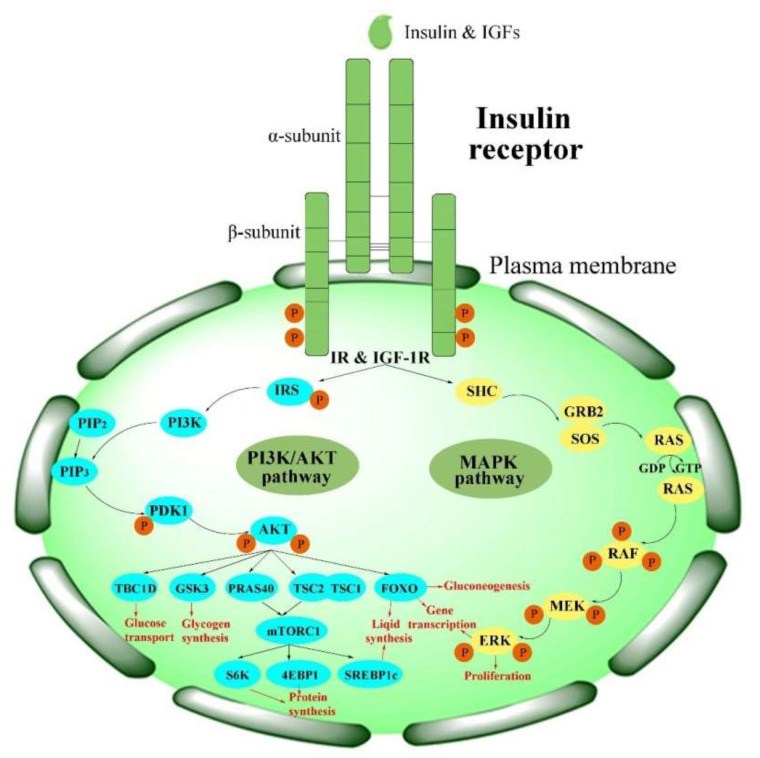
Activation of the IR in insulin signaling pathways. PI3K/AKT pathways: phosphatidylinositol-3-kinase signaling pathways; MAPK pathway: mitogen-activated protein kinase pathway.

Physiologically, the function of the IR is activated in the insulin/IGF-1-like signal (IIS) pathway by the ligand [2]. The IIS pathway is commonly known as a significant nutrient-dependent endocrine pathway and regulates numerous physiological processes, such as metabolism, growth and development, and so on [6]. In the IIS pathway, the IR regulates two primary cell-signaling cascades (Figure 2) [53]: the phosphatidylinositol-3-kinase (PI3K)/AKT signaling pathway and the mitogen-activated protein kinase (MAPK) pathway (extracellular-signal regulated kinase signaling pathway (ERK)) [53,54,55,56]. The PI3K/AKT pathway is primarily responsible for controlling metabolic processes such as glucose transportation and the synthesis of lipids, proteins, and glycogen. In contrast, the MAPK pathway is primarily related to the mitogenic effects of insulin and is mainly responsible for cell growth and proliferation [56,57].

The major upstream factors of the IIS pathway are various insulin-like peptides (ILPs). Based on primary structure and receptor binding preferences, these ILPs can be subdivided into insulin, insulin-related growth factors (IGFs, including IGF-I and IGF-II) in mammals, and ILPs in insects [58]. Insulin is a peptide hormone secreted by pancreas β-cells and is one of the most conserved molecules in animals [59,60]. IGFs are peptides that have a homology of 40–80% with insulin. In humans, both insulin and IGFs can bind to the IR on the cell surface and functionally mediate cellular proliferation and differentiation, lipid metabolism, glucose homeostasis, and DNA synthesis [60,61]. Meanwhile, in insects, ILPs are the most general growth-promotion signaling factors [62,63,64,65], and evidence has suggested that ILPs are homologues of human insulin [6,66]. Insulin is the major regulatory factor in humans, but various ILPs have been identified in different insect species, ranging from one—in the *Nevada dampwood* termite, *Zootermopsis nevadensis* (Hagen)—to more than 40—in the silkworm, *Bombyx mori* L. [60].

ILPs first phosphorylate the IR and then activate IR signaling. The tyrosine-phosphorylated IR, in return, recruits and phosphorylates other intracellular adaptor proteins, such as IR substrate (IRS) proteins and several other substrates, including Src homology 2 domain-containing (SHC), Grb2-associated binder (GAB), APS (SHB2), and Cbl, at several tyrosine residues [56,67]. There are six isoforms (IRS1–6) in the IRS family [68]; among these, IRS1 and IRS2 are the main isoforms [68,69]. These proteins mediate the association with the Src homology 2 (SH2) domains and lead to initiation of the PI3K/AKT pathway, as well as activation of the downstream phosphoinositide-dependent kinase (PDK1) and protein kinase B (PKB, also called AKT) [4]. Phosphorylation of the IR triggers the activation of cellular signaling pathways, which play different roles in human beings and insects.

## 3. Functions of the IR

### 3.1. The Functions of the IR in Human Beings

In humans, the IR plays a crucial role in whole-body nutrient homeostasis and in various diseases, such as AD [8], T2DM [4,9,10], obesity [70], atherosclerosis [31], multiple cancers [11,12,13], and cardiovascular disease [71], as well as neurodegenerative disorders [14], metabolic syndrome [15] and polycystic ovary syndrome [72]. Thus, it is necessary to understand the cellular expression and the functions of the IR in order to propose new treatment concepts and to develop novel drugs. 

The IR mediates whole-body nutrient homeostasis and is expressed ubiquitously through the classic insulin-responsive targets in the liver, muscle, and adipose tissue [3]. Recent work has demonstrated that the IR is distributed in both dendritic shafts and spines in living hippocampal brain neurons [73]. Knockout of the IR resulted in many impaired target organs. Hepatic deletion of the IR led to hyperglycemia, disorders in fatty acid metabolism, and an increase in the expression of fatty acid oxidation enzymes [74]. In mucosal epithelial cells, the IR interacts with the voltage-dependent anion channel-1 (VDAC1) in mitochondria. Knockdown of the IR gene triggered robust mitochondrial fragmentation and altered polarization [75], while knockout of the β-cell IR gene led to impaired insulin secretion [76]. Additionally, missense mutations of the IR may cause severe inherited insulin resistance syndromes [77].

The IR is a cell-surface receptor translocating to the nucleus, and is associated strongly with RNA polymerase II in the chromatin [78]. In the cell, host cell factor-1 (HCF-1) acts as a transcriptional coregulator functionally mediating the binding of the IR to specific sites located in the gene promoters [79]. HCF-1 mediates the association between the IR and DNA. HCF-1 binds to DNA indirectly through DNA sequence-specific transcription factors, and then forms a complex with the IR and Thanatos-associated protein domain-containing protein 11 (THAP11) in the chromatin. Knockdown of HCF-1 can inhibit the binding ability of the IR to the promotors [79]. Another study indicated that the mRNA and protein levels of the IR were obviously reduced in the subcutaneous and visceral adipose tissue of women with gestational diabetes mellitus (GDMs) [80]. The decrease in IR mRNA was accompanied by a decrease in methylation levels of the IR promoter [80]. This phenomenon has also been observed in the hypothalamus [81]. The methylation degree of the IR nuclear factor I (IRNF-I) binding site within the IR promoter was dramatically inversely correlated with the gene level of the IR. These findings have opened a new avenue for further studies on the functions and mechanisms of the IR. More studies focusing on demonstrating whether epigenetic modifications in the IR sequence impact IR expression of the IR are needed [82].

### 3.2. The Functions of the IR in Insects

Studies of the IR in human beings have raised interest in the functions of the IR in insects and the consequent possibility for the development of new IR-targeting insecticides with high efficiency and low toxicity. 

In insects, multiple functions of the IR have been revealed [83]. The IR is well-known to be implicated—either directly or by crosstalk with other major hormones such as juvenile hormone (JH) and ecdysteroids (especially 20-hydroxyecdysone, 20E)—in post-embryonic development [84,85], nutrition-based phenotypic plasticity and body size control [86,87], reproduction and diapause [55], and circadian rhythmicity and behaviors [88,89,90]. The IR is also indispensable in insect photoperiodism, lifespan, and aging due to its relation to metabolism and growth [25,91,92,93]. Overall, studies have indicated that the IR is indispensable in insect growth [94], development and reproduction [95,96], polymorphism [24], lifespan [97], and oviposition [98]. Therefore, the IR represents an important target for the management of pests and parasites. 

#### 3.2.1. Gene Organization of the IR in Insects

The first invertebrate IR was purified from the extracts of adult *Drosophila melanogaster* based on its binding to radiolabeled insulin [99]. Different from the IR in human beings, the IR presents various types in insects (named InR). For example, only one type of InR has been reported in insects such as the fruit fly *Drosophila melanogaster* and the silkworm *Bombyx mori*, while evidence has also indicated that two types of InRs (InR1 and InR2) exist in other insects, such as the honeybee *Apis mellifera* and the brown planthopper *Nilaparvata lugens*. Interestingly, three InRs have been discovered in blattodeans, termites, and the firebug, *Pyrrhocoris apterus* [92,100]. In termites, InR1 and InR3 levels remained stable, while InR2 was differentially expressed between workers and reproductive females [100]. This indicates that the InR2 level is closely related to caste. However, in German cockroaches, there was no difference in the expression of InR2 between larvae and adult females [100].

The IR gene of *Drosophila* (DInR) has been used as a model for the study of the IR. The DInR is secreted from neurosecretory cells in the brain [29,60] and other tissues, such as the midgut, fat body, and imaginal discs [6]. The DInR exhibits high levels in the central nervous systems of larvae and adult fruit flies. It is necessary for the formation of aversive olfactory learning, associative long-term memory, and intermediate-term memory in aged flies [101,102]. Unlike previously published data, recent evidence has indicated that the DInR in the mushroom body Kenyon cells suppress the formation of anesthesia-resistant memory and stimulated the formation of a longer-lasting memory in larval *Drosophila* [90]. The DInR mediates and encodes eight insulin-like peptides (DILPs 1–8) [103,104,105]. Among these DILPs, DILPs 2, 3, and 5 are expressed in insulin-producing cells (IPCs), then released into the hemolymph [104]. DILP 6 is structurally and functionally similar to IGF, while DILP 8 is a homolog of relaxin. The overexpression of any of DILPs 1–8 led to an increase in larval size [6]. The interaction of DILPs with the IR is highly conserved through the complex and versatile IIS pathway.

#### 3.2.2. The Effect of Silencing or Knockdown of IR Genes in Insects

The silencing of IR genes produces a variety of phenotypes in insects. In the incomplete metamorphosis brown citrus aphid, *Aphis citricidus* (Kirkaldy), two IR genes were observed, *AcInR1* and *AcInR* [106]. *AcInR1* increased during the transition from nymph to adult in alate aphids, while *AcInR2* had the highest expression level in second instar nymphs. The silencing of *AcInR1* or *AcInR2* by RNAi resulted in 73% or 60%, respectively, of aphids having problems in the transition from nymph to a normal adult. The co-silencing of *AcInR1* and *AcInR2* genes led to 87% of aphids having problems in the transition from nymph to normal adult and 62% dead nymphs. Therefore, *AcInR1* and *AcInR2* are essential for a successful nymph–adult transition in alate aphids. In the complete metamorphosis insect red flour beetle, *Tribolium castaneum* Herbst, two IR genes have also been observed, *TcInR1* and *TcInR2*. *TcInR1* presented a high level in the late adult stage and the early pupal stage, whereas *TcInR2* was strongly expressed during the late larval stage. The silencing of *TcInR1* led to a death rate of 100%, while the silencing of *TcInR2* caused a death rate of 42% in larval and parental *T. castaneum* [95]. For the legume pod borer *Maruca vitrata* (Fabricius), InR-silenced insects were inhibited at the larval–pupal stage and then died [107]. 

The silencing of IR genes might relate to the different development stages in insects. However, knockdown of the corresponding IR genes led to various results in different insects. In *T. castaneum*, the knockdown of *TcInRs* reduced food consumption and decreased larval weight and size [94]. In the rhinoceros beetle *Trypoxylus dichotomus* L., knockdown of the IR gene caused a dramatic reduction in the length of the adult horn, with a slight reduction in wing and genital size [86]. In female mosquitoes, such as *Aedes aegypti* (Linnaeus) [108], *Culex quinquefasciatus* Say [109], *Culex pipiens* Linnaeus [110], and *Anopheles gambiae* Giles [111], knockdown of the IR gene led to smaller ovaries and marked reductions in oviposition. In the female blood-feeding bug *Rhodnius prolixus* Stål, knockdown of RhoprInR also decreased the formation of eggs [112]. The impairment of IR genes also resulted in negative impacts on the fecundity, immune response, and blood digestion of *A. aegypti* [108,112].

#### 3.2.3. Polyphenism Is Adjusted by the IR in Insects

Polyphenism is a form of developmental plasticity, and is a successful strategy adopted by organisms to adapt to changing environments [113]. Examples in insects are caste differentiation in eusocial insects, wing polyphenism in planthoppers and aphids, sexual dimorphism in the brown planthopper and fruit fly, and seasonal polyphenism of butterflies [114]. Evidence indicates that insect polyphenism is connected to the multiplicity of the IRs.

Caste differentiation: Social insects, such as bees and ants, are ideal models for investigating caste differentiation mechanisms due to the intraspecific variations in their sexual dimorphism [115]. In the honeybee *Apis mellifera* and the fire ant *Solenopsis invicta*, the IR has been shown to regulate caste differentiation [116,117,118]. In critical stages of caste development of the honeybee, the IR genes *AmInR-1* and *AmInR-2* are primarily expressed in the second instar of queen larvae and sharply declined in the third and fourth instars of queen larvae, while little change is observed in worker bees [117]. *AmInR-2* had the highest level in the queen larvae after hatching [116,119]. Similarly, in fire ants, the two *SiInR*s were differently expressed in the early development of the queen and worker larvae ants [120]. The expression of *SiInR-1* in eggs was the highest except for the fourth instar larvae of workers, virgin queens, and males, while the expression of *SiInR-2* in eggs was significantly higher than that in other stages [118]. Expression of both of the corresponding receptors was significantly higher in virgin queens and males compared to that in adult workers. 

Caste differentiation in cockroaches and termites has been related to InR2 and InR1, while InR3 had no impact [100]. Target of rapamycin (TOR), epidermal growth factor receptor (Egfr), juvenile hormone (JH), and vitellogenin (Vg) are also involved in insect caste determination [120]. In queen predetermined larvae, the knockdown of genes of the IRs, TOR, and Egfr resulted in more workers [121,122]. 

Wing polyphenism: Wing polyphenism is an evolutionarily successful feature that enables insects to adapt to environmental changes [123]. This phenomenon is commonly observed in a wide range of wing polymorphic insects, such as the brown planthopper *Nilaparvata lugens* (Stal) and the pea aphid *Acyrthosiphon pisum* (Harris) [106,123]. The interactions between the IIS pathway, ecdysone, and JH signaling are involved in wing dimorphism [124]. The brown planthopper has been well-studied due to its incomplete metamorphosis leading to two wing morphs: long wing (LW) and short wing (SW) [124,125,126,127,128]. The InRs (InR1 and InR2) play antagonistic roles to determine long versus short wing development by regulating forkhead transcription factor subgroup O (FoxO) activity [123,125]. Naturally, the activation of InR1 induces the formation of the long wing through the PI3K–AKT–FoxO signaling cascade, while a high level of InR2 in wing buds leads to short wings [127]. The *decapentaplegic* (dpp) gene can respond to the switch genes (*NlInR1* and *NlInR2*) and participates in wing morph development through a dose-dependent response [126]. Unlike the brown planthopper, three InRs have been found in the firebug *Pyrrhocoris apterus* (Linnaeus). Two gene clusters are involved in the wing polyphenism of the firebug. It has been observed that a pattern of Cluster I and II of the InRs impacts wing development, in contrast to that postulated in planthoppers, suggesting independent establishment of IIS in the control of wing development [92].

Sexual dimorphism: Recent research has shown that knockdown of the sex determination gene Transformer-2 in adult *N. lugens* led to long wing female offspring [129]. This suggests the existence of crosstalk between sex differentiation and wing dimorphism during embryonic stages. For the synthesis of JH, the corpus allatum (ca) is the key gland, while 3-hydroxy-3-methylglutargyl CoA reductase (HMGCR) is also an important enzyme. The InR in the ca and the enzyme HMGCR have been implicated in the control of sexual dimorphism in *Drosophila* [130,131]. The knockdown of the InR gene in the ca oppressed the gene encoding of HMGCR, resulting in interruption of sexual differentiation and the emergence of dwarf flies [131].

In brief, the InR gene plays important roles in regulating caste differentiation, wing polyphenism, and sexual dimorphism in insects, and thus is significant for future studies on the inhibition, knockdown, or modulation of the InR in insects.

## 4. Pharmacological and Physiological Modulators of IR Activation

The IR has recently made its mark as an attractive therapeutic target for a variety of cancers and diabetes in humans due to its overexpression in various cancers [13,32]. Thus, agonists and antagonists of the IR that selectively activate or inhibit the IIS pathway may have considerable value in providing promising drugs for human diseases [4,132]. However, in contrast to the diverse regulators of insulin, only a handful of modulators have been discovered for the IR [133]. In the following, we provide a summary of existing IR modulators in order to provide an enriched yet challenging prospect for novel therapeutic approaches.

### 4.1. Insulin and Its Analogs

As the upstream regulator of the IR, insulin has been studied for more than 100 years since first being reported in 1921. Insulin has an autocrine/paracrine regulatory role on its own signaling system. Binding of insulin to the activated IR leads to negative cooperativity and pleiotropic effects on the IIS pathway [134]. Insulin analogs can be classified as short- or long-acting based on their pharmacokinetics and general principle of protein folding and assembly [32] (Table 2). 

Short-acting insulin analogs, such as insulin lispro, aspart, glulisine, and AspB10, have a faster onset and shorter duration of action, while long-acting analogs, such as glargine, detemir, and degludec, have a more stable insulin action profile with longer duration than human insulin [135]. Both can interact with the IR and the insulin-like growth factor I receptor (IGF-1R), with varying binding affinities and dissociation rates [32]. Therefore, more and more speculations have been raised that insulin analogs could be used for the treatment of T2DM [136,137]. 

### 4.2. Insulin-Mimetic Peptides

Many insulin-mimetic peptides, such as Site 1 insulin-mimetic peptide S371, Site 2 peptide S446, single-chain Site 2–Site 1 optimized peptide S519, Site 2–Site 1 combination peptide S597 and S661, and Site 1–2 peptide S961, have been obtained to selectively function as agonists or antagonists to modulate the IR [138,139] (Table 2). S371 is part of the potent receptor S519, which exhibits agonist activity and competes with the αCT for binding to the L1 domain of the IR. S597 is a further optimized agonist peptide of S519, binding to the IR to adjust its phosphorylation. Furthermore, S661 and S961 are antagonists of the IR [140,141]. Interestingly, S961 is a special example, showing both agonist and antagonist effects on the human IR [140,142]. 

### 4.3. Antibodies

Antibodies are agonists of the IR. Many antibodies (non-insulin ligands) have been reported to modulate the activity and function of the IR [4] (Table 2). The first generation of IR autoantibodies was distinguished in the control of some human diseases [143]. They displayed confirmed selectivity, pathophysiological sensitivity, and more excellent safety than the traditional regulating agents [133]. 

XMetA is a fully human IgG2a monoclonal antibody that is a direct agonist of the IR [144,145]. XMetS is another allosteric IR agonist antibody that has little effect on the IR without the presence of insulin [146]. The combined use of insulin and XMetS modulated the insulin binding affinity and IR autophosphorylation more strongly and positively than insulin and IgG by nearly 18-fold and 14-fold, respectively [145]. In addition to these positive antibodies, many antibodies act as antagonists. XMetD (also known as X358 or XOMA 358) is a negative modulator [147]. XMetD reduced the insulin binding affinity about 3-fold and decreased the sensitivity of insulin-stimulated IR autophosphorylation about 40-fold, and was the first monoclonal antibody to be advanced into human clinical studies [147,148]. 

Hinke (2018) recently reported a novel allosteric agonist IR monoclonal antibody, IRAB-A, which exhibited sensitizing effects on IR and Akt phosphorylation based on cell assays [132]. Further, another novel antagonist antibody, IRAB-B, has been found, which can specifically bind to the IR with nanomolar affinity, and thus can induce rapid and continuous insulin resistance [149]. 

Interestingly, a new method (anti-idiotypic antibody strategy) by hybridoma technology for the development of IR antagonists has led to the anti-idiotypic antibody AK98. AK98 has exhibited good antagonistic activity against the IR in a tumor cell model. In particular, AK98 presented a dose-dependent effect in the inhibition of IR-mediated signaling pathways [23].

### 4.4. Allosteric Aptamers

Another type of selective allosteric activator of the IR is aptamers. Aptamers are single-strand oligonucleotides (DNA or RNA) from random oligonucleotide libraries [150]. They can specifically and strongly bind to the target with high affinity due to their unique 3D structures [151]. Studies have reported on various aptamers (Table 2), such as IR-A48 [152], IR-A43 [153], IR-A62 [154], and GL56 [155]. IR-A48 is an agonistic aptamer that binds to and then activates the IR [152]. IR-A43 is a sensitizing aptamer that effectively binds to the IR and enhances insulin sensitivity [153,156]. IR-A62 acts as a biased agonist that binds to the extracellular domain of the IR and preferentially induces Y1150 monophosphorylation of IR [154].

In contrast to IR-A48, IR-A43, and IR-A62, GL56 is a nuclease-resistant RNA aptamer that specifically recognizes the IR and acts as a neutralizing ligand to inhibit IR activity and IR-dependent signaling [155]. GL56 provides a novel avenue for future research on the clinical therapy of IR-dependent cancers. 

### 4.5. Proteins

#### 4.5.1. Adaptor Proteins and Regulatory Proteins

Some cytoplasmic adaptor proteins (Table 2), such as growth factor receptor-bound protein 10 (GRB10), growth factor receptor-bound protein 14 (GRB14), and suppressors of cytokine signaling proteins (SOCS; in particular, SOCS1 and SOCS3), can inhibit IR activity by acting as pseudosubstrates of IR kinase [157]. Further information can be found in the paper by Haeusler (2018) [3]. 

New proteins have been reported to regulate the IR, such as SH2 domain-containing adaptor protein (SH2B1). SH2B1 can directly bind to the IR and insulin receptor substrate (IRS), and enhances the catalytic activity of the IR and suppresses the dephosphorylation of the IR as an endogenous insulin sensitizer [157]. GRP78 (78 kDa glucose-regulated protein) is a multi-functional chaperone that promotes the phosphorylation and activation of IGF-1R. Thus, GRP78 inhibitors could inhibit IGF-1R signaling in hepatoma cells [158]. Additionally, the expression of the IR in target cells is also influenced by factors such as the sorting-related receptor with type A repeats (SORLA) protein, which acts as a sorting factor for the IR, enhancing its surface expression [70].

#### 4.5.2. Membrane Proteins

Caveolins are lipid-raft-associated integral membrane proteins [159]. Proteins in the caveolin family are encoded by three genes and consist of six known caveolin subtypes: caveolin-1α, caveolin-1β, caveolin-2α, caveolin-2β, caveolin-2γ, and caveolin-3 [160]. Of the six known caveolins, caveolin-2α (Cav-2α) is a positive regulator of insulin signaling [161], while caveolin-2β (Cav-2β) is a negative regulator [162]. Cav-2α gathers the IR and initiates the IRS-1 signaling system [161,163]. Cav-2β desensitizes the IR through the dephosphorylation of protein-tyrosine phosphatase 1B (PTP1B), followed by IR endocytosis and lysosomal degradation, thus resulting in insulin resistance [162,163].

Apolipoprotein E (ApoE) is a glycoprotein consisting of 299 amino acids. ApoE has three isoforms; namely, E2, E3, and E4 [164]. Among the ApoE genes, the ε4 allele (*apoE4*) is the most substantial genetic risk factor for AD, compared with the ε2 allele (*apoE2*) and ε3 allele (*apoE3*). In primary neurons, ApoE4 specifically binds to the IR and reduces its transportation by trapping it in the endosomes [165]; hence, its binding negatively affects the IIS pathway and insulin-evoked mitochondrial respiration and glycolysis due to disruption of the interaction between insulin and the IR in an isoform-dependent manner [166,167]. 

### 4.6. Other Pharmacological and Physiological Modulators

In addition to the IR regulators mentioned above, some biomolecules, such as proteoglycan, peptides, enzymes, and some drug mixtures, also regulate the IR. In detail, glypican-4 (Gpc4) is a member of the glycosylphosphatidylinositol (GPI)-anchored heparan sulfate proteoglycan family and binds to the IR and potentiates the IIS pathway. Gpc4 also promotes adipocyte differentiation. Impairment of Gpc4 led to a reduction in IR activation and suppression of adipocyte differentiation in vitro [168]. 

The 9-amino-acid-residue peptide (mcIRBP-9) is a peptide from the bitter gourd *Momordica charantia* L. that targets the IR and enhances the activity of IR kinase [169]. Another peptide is visfatin, which binds to the IR at a site distinct from that of insulin and causes hypoglycemia by reducing glucose release from liver cells and stimulating glucose utilization in adipocytes and myocytes [170].

Sphingomyelin phosphodiesterase acid-like 3b (SMPDL3b) is a lipid-raft enzyme that impairs IR-B-dependent insulin signaling by interfering with IR binding to Cav-1 in the plasma membrane (PM) [171]. Protein tyrosine phosphatase 1B (PTP1B) and protein kinase Cε (PKCε) are also negatively involved in regulation of the IR. PTP1B dephosphorylates the IR, leading to its deactivation. Studies in PTP1B knockout mice have revealed improved insulin sensitivity and IR phosphorylation in muscle and liver [172,173]. PKCε impairs IR autophosphorylation by phosphorylating its substrate, Thr^1160^, in the functionally critical IR kinase activation loop [174]. 

Aroclor 1254 is a commercial polychlorinated biphenyl (PCB) mixture that inhibits the IR signaling pathway, including the IR, IRS, PI3K-AKT, and PKB, in skeletal muscle and the liver [175]. Subetta is a drug that releases active forms of antibodies to the IR β-subunit and endothelial nitric oxide synthase. Subetta increased phosphorylation of the β-subunit of IR with or without insulin in vitro [176].

**Table 2:** Pharmacological and physiological modulators of IR activation.

Classification	Modulators	IR Modulation Mechanism	Model Organisms or Cells	Side Effects	References
Insulin and insulin analogs	insulin and IGFs,	ligand-induced internalization and degradation of the IR	human	tissue irritation, abscesses, allergic edema, weight gain, risk of congestive heart failure	[3]
	lispro,				[32,135]
	aspart,				
	glulisine,				
	aspb10,				
	detemir,				
	largine,				
	degludec,				
	ILPs	ligand	insects	-	[103]
Insulin-mimetic peptides	S371, S446	disrupts the primary insulin binding site of the IR	mice	-	[138]
				-	
	S519(agonist)			-	
	S597 (partial agonist)	receptor activation	IR-transfected L6 myoblasts	-	[138,177]
	S661	antagonist of the IR	rat adipocytes	-	[140]
	S961(agonist/ antagonist)	↓IR, blocks expression of the IR without insulin	breast cancer cells	-	[141,178]
Antibodies	XMetA (partial agonist)	↑IR autophosphorylation (EC_50_:1.3 nmol/L); ↑Akt phosphorylation (EC_50_: 1.1 nmol/L)	CHO-hINSR cells (in vitro); diabetic mice (in vivo)	-	[144,145]
	XmetS (agonist)	↑binding affinity with IR; ↑IR autophosphorylation (insulin-dependent); ↑Akt phosphorylation	MCF-7 human breast cancer (in vitro); mouse models of insulin-resistant diabetes (in vivo)	-	[146]
	XmetD (X358) (antagonist)	↓autophosphorylation of IR (interacte with IR); ↓phosphorylation of Akt and Erk	adult male CHO-hINSR cells; L6 muscle cells; COLO-205 human colon cancer cells; hyperinsulinemic hypoglycemia mice	-	[147]
			healthy adult	insulin resistance (3 d wherein X358-imparted)	[148]
	IRAB-A (agonist/sensitizer)	↓off-rate of insulin from the IR (stabilizes insulin binding)	diet-induced obese C57 mice	-	[132]
	IRAB-B (antagonist)	↓IR phosphorylation (binds to IR)	C57BL/6N mice	-	[149]
	AK98 (antagonist)	competes with insulin (bind to IR) ↓IR expression levels	tumor cell (MCF-7)	-	[23]
Aptamers	IR-A48 (partial agonist) (IR Tyr1150),	↑IR autophosphorylation (allosteric binds and activates the IR, but not IGF-1R)	HEK293 and 3T3-L1 cells; Rat-1 cells overexpressing human IR (Rat-1/hIR)	-	[152]
	IR-A43 (sensitizer)	binds to the allosteric site of IR; ↑insulin bind to IR	-	-	[153]
	IR-A62 (agonist and activator)	↑insulin binding and Y1150; monophosphorylation of the IR (low concentrations); ↓insulin binding and IR phosphorylation (high concentrations)	C57BL/6 mice; Rat-1 cells overexpressing human IR (Rat-1/hIR); 3T3-L1 and MCF-7 breast cancer cells	-	[154]
	GL56 (inhibitor)	specifically recognizes the IR; ↓IR phosphorylation; ↓phosphorylation of AKT, ERK1/2 and IRS1	U87MG; glioblastoma cancer cells	-	[155]
Proteins	GRB10/14,	↓activity of the IR as a pseudosubstrate of the IR-TK	mice	-	[157]
	SOCS1/3,		mice	-	[3]
	GRP78 (IGF-1R)	↑IGF-1R phosphorylation and activation	hepatoma cells	-	[158]
	SH2B1	↑IR and IRS1 phosphorylation;↑Akt and Erk activation	CHO–IR, 3T3L1, NIH3T3, and HEK293 cells; mice	-	[157]
	SORLA	↑IR surface expression (redirects internalized IR from endosomes to PM)	mouse with loss of function/tissue -specific over- expression of SORLA; obese human subjects	-	[70]
	Cav-2α	↑IRS-1 recruitment and association with IR (a substrate of IR tyrosine kinase)	Hirc-B cells, HEK293T cells, 3T3L1 preadipocytes or adipocytes	-	[161]
	Cav-2β	desensitization of the IR; ↑IR-TK inactivation via dephospho-rylation by PTP1B and internalization via dynamin- 2-dependent endocytosis	HEK293T cells, 3T3-L1 preadipocytes (ATCC, CL-173)	-	[162]
	ApoE	interacts with the IR, interfering with insulin binding; ↓insulin–IR interaction and impairs IR trafficking	human ApoE -targeted replacement mice	-	[164,165]
Others	Glypican-4	interacts with the IR, causing ↑IR signaling	visceral and subcutaneous adipose tissue/3T3-L1 preadipocytes	-	[168]
	mcIRBP-9	↑IR kinase activity; ↑phosphorylation of IR; ↑translocation of GLUT4; ↑uptake of glucose	3T3-L1 preadipocytes; type 1 diabetic mice; type 2 diabetic mice (*db*/*db* mice)	-	[169]
	Visfatin	binds to the IR site	clonal mouse pancreatic β-cell; β-TC6 cell line (BTC) cells	-	[170]
	SMPDL3b	interferes with the IR isoforms binding to caveolin1 in the PM	podocytes in DKD	-	[171]
	PTP1B	dephosphorylates the IR, causing deactivation	mice	novel therapeutic strategy for T2DM	[172]
	PKCε	phosphorylates the IR, blocking IR autophosphorylation	Insr^T1150A^ mice	improves NAFLD diagnostic screening for the early identification of patients at risk for T2D	[174]
	Aroclor 1254	inhibits the expression of the IR	male C57BL/6 mice/skeletal muscle & liver	_	[175]
	Subetta	increases IR β-subunit phosphorylation	human preadipocytes	_	[176]
	BACE1	cleaves the IR ECD and decreases the amount of mature IR	mouse models of diabetes (db/db) and impaired glucose tolerance (HFD mice)	_	[21]

ILPs: insulin-like peptides; GRB: growth factor receptor-bound protein; SOCS: suppressor of cytokine signaling; SH2B1: SH2 domain-containing adaptor protein; SORLA protein, sorting-related receptor with type A repeats; Cav-2α: caveolin-2α; Cav-2β: caveolin-2β; ApoE: apolipoprotein E; mcIRBP-9: 9-amino-acid-residue peptide; SMPDL3b, sphingomyelin phosphodiesterase acid-like 3b; PTP1B: protein-tyrosine phosphatase 1B; PKCε: protein kinase Cε; NAFLD: nonalcoholic fatty liver disease; BACE1, β-site amyloid precursor protein cleaving enzyme 1; PM, plasma membrane; Hirc-B, human IR-overexpressed rat 1 fibroblast cells; CNS: central nervous system; DKD, diabetic kidney disease; CHO, Chinese hamster ovary; Insr^T1150A^ mice, C57BL/6J mice harboring a threonine-to-alanine mutation at the homologous residue Thr^1150^.

### 4.7. Mechanism of the Pharmacological and Physiological Modulators of IR Activation

**Figure 3:**
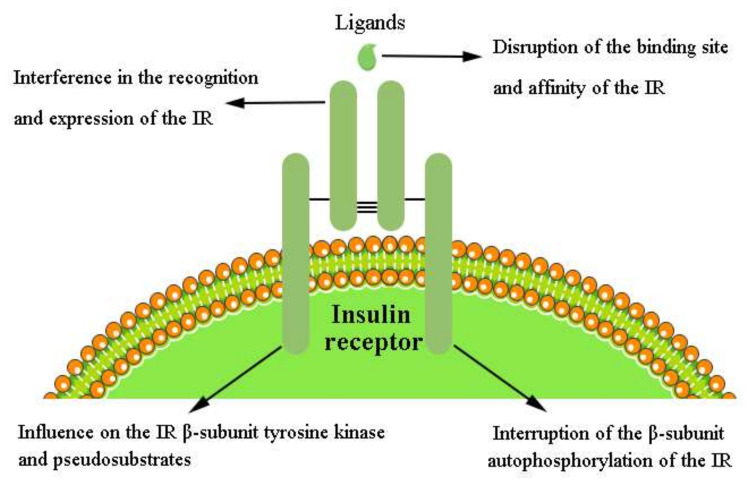
Modulation mechanism of pharmacological and physiological modulators of IR activation.

In conclusion, insulin and insulin analogs, insulin-mimetic peptides, monoclonal antibodies, aptamers, proteins, proteoglycan, peptides, enzymes, and some drug mixtures are the main pharmacological and physiological modulators of the IR (Table 2). In vitro evidence has indicated that the IR is regulated through diverse and complex mechanisms. Herein, we classify these mechanisms into four main types [68] (Figure 3):

#### 4.7.1. Disruption of the Binding Site and Affinity of the IR

The IR is a heavily glycosylated membrane-spanning protein. Therefore, the insulin binding site and affinity are crucial for modulation of the IR [146]. Disruption of the primary binding site of the IR is a prominent positive modulation [177]. In contrast, the primary negative modulation of IR is through interference with the interaction of insulin and the IR (e.g., ApoE). 

#### 4.7.2. Interference in the Recognition and Expression of the IR in the IIS Pathway

Normally, insulin binds to the IR and, in turn, activates the insulin-induced (IIS) pathway. Therefore, the ligand itself is undoubtedly the crucial negative regulator of its signaling [179]. Adjustment of the recognition and expression of the IR by modulators could positively regulate IR signaling. On the contrary, insulin, picropodophyllin (a specific IGF-IR inhibitor that completely abolishes insulin- and IGF-II-induced migration in IRB), Cav-2β, GL56, and Aroclor 1254 are negative regulators of the IR that inhibit IR-dependent signaling.

#### 4.7.3. Interruption of β-Subunit Autophosphorylation of the IR

After insulin binding, the IR undergoes conformational changes, resulting in autophosphorylation of tyrosine residues in the intracellular β-subunits of IR (IR-β) to activate the intrinsic tyrosine kinase in IR-β [180]. The activated IR-β triggers phosphorylation of downstream signaling molecules. IR autophosphorylation has been regarded as a hallmark of IR activation and plays a determinative role in the modulation of receptor-associated kinase activity towards exogenous substrates [181]. Recent work has demonstrated that IR autophosphorylation is a stepwise process, where symmetric mono-phosphorylation of the Tyr^1150^ residue (m-pY1150) is a key characteristic of biased agonists to the IR [50]. The m-pY1150 is necessary for the juxtamembrane (JM) domain to release the kinase and Tyr residues for phosphorylation [50]. Additionally, m-pY1150 determines the substrate specificity of the IR kinase, and the induction of m-pY1150 is an essential step for full IR activation. Hence, stimulation of m-pY1150 induction and IR autophosphorylation activation are favorable regulatory factors for the IIS pathway. However, dephosphorylation and inhibition of autophosphorylation are negative regulatory factors for the IR; the effect of protein tyrosine phosphatase 1B (PTP1B) on the IR is an example of this.

#### 4.7.4. Influence on the IR β-Subunit Tyrosine Kinase and Pseudosubstrates

Activators of IR–tyrosine kinase (IR-TK) could enhance the activity of the kinase after autophosphorylation of the β-subunit of the IR. The adaptor proteins (GRB and SOCS) are inhibitors of the IR and contain two different domains: one being a peptide region binding to the IR substrate-binding cleft, and the other being the SH2 domain that binds to and occupies the IR phosphotyrosine site. These protect the IR phosphotyrosines from phosphatases, thus enhancing IR phosphorylation. These GRBs and SOCS act as pseudosubstrates of IR kinase and inhibit IRS binding [3].

## 5. Nonpeptide Small Molecule Modulators of the IR

**Figure 4:**
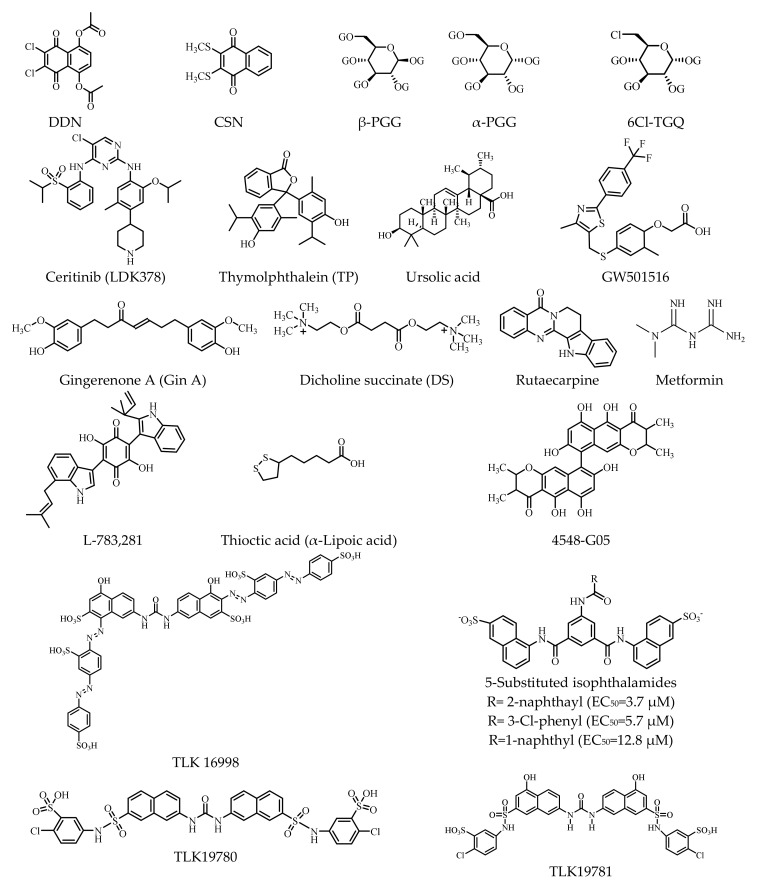

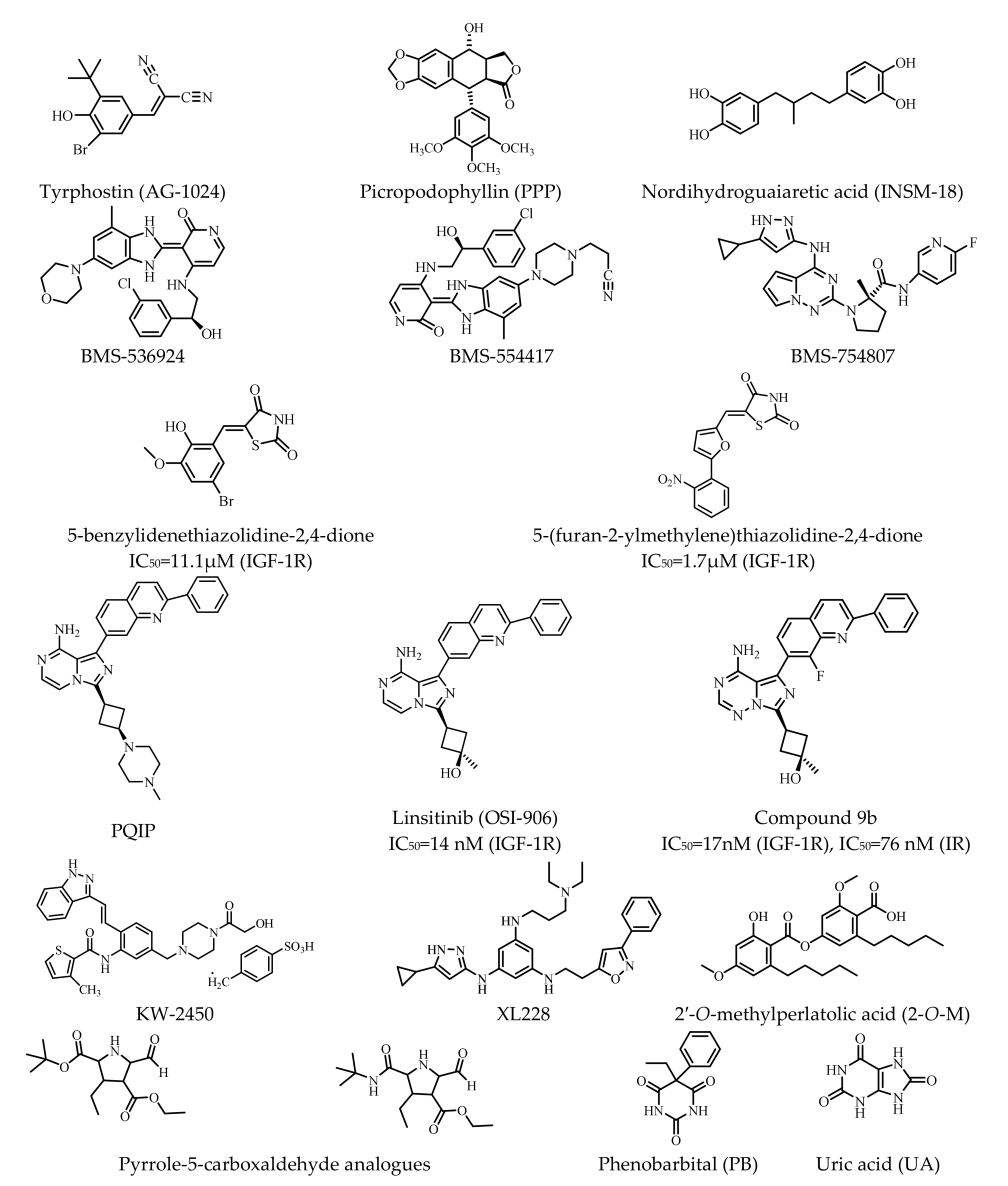
Structure of small-molecule positive modulators of the IR.

Small molecules with molecular weights below 500 Da have recently received more attention due to their high potential for specificity based on the physiological abnormalities of human diseases [182]. Therefore, they are considered promising in the development of new drugs. Basically, they can be classified into three groups: (1) small-molecule inducers of IR autophosphorylation, (2) modulators of the tyrosine kinase domain of the IR β-subunit, and (3) regulators of the IR and IGF-1R (Table 3). The structures of these small molecules are presented in Figure 4.

### 5.1. Small Molecule Inducers of IR Autophosphorylation

Once insulin binds to the α-subunit of the IR, autophosphorylation of tyrosine residues of the β-subunit occurs. Hence, compounds—such as thymolphthalein (TP, an agonist), dicholine succinate (DS, a mitochondrial complex II substrate), and GW501516 (2-methyl-4-((4-methyl-2-(4-trifluoromethylphenyl)-1,3-thiazol-5-yl)-methylsulfanyl)phenoxy-acetic acid, a PPAR β/δ agonist), as well as some other naphthoquinone derivatives and natural chemicals from plants—that stimulate the phosphorylation of the IR present one pattern of modulation [183,184,185,186,187] (Table 3). 

Presently, the naphthoquinone derivatives include DDN (5,8-diacetyloxy-2,3-dichloro-1,4-naphthoquinone), CSN (2,3-bismethylsulfanyl-1,4-naphthoquinone), and ceritinib (LDK378, 5-chloro-N2-(2-isopropoxy-5-methyl-4-(piperidin-4-yl)phenyl)-N4-(2-(isopropylsulfonyl) phenyl) pyrimidine-2,4-diamine) [188,189,190], while the natural chemicals from plants consist of tannin chemicals such as α-PGG (α-penta-galloyl-glucose), β-PGG (β-penta-galloyl-glucose), and 6Cl-TGQ (6-chloro-6-deoxy-1,2,3,4-tetra-*O*-galloyl-α-D-glucopyranose) [191,192,193].

Additionally, adenosine (5′-Se-methyl-5′-seleno-,2′,3′-diacetate) [non-peptidyl compound 43 (NPC43)] and a plant-derived polyphenol chemical, gingerenone A (Gin A), have also been reported as inducers of IR autophosphorylation [194,195]. In addition to these tannin derivatives and polyphenols, polycyclic natural products such as ursolic acid can also enhance the autophosphorylation of the β-subunit of the IR and subsequently affect the PI3K pathway downstream [196,197]. Rutaecarpine is extremely significant and is regarded as an analog of metformin (dimethyl biguanide, the first-line drug for the treatment of T2DM) [198,199,200] (Table 3).

### 5.2. Modulators of the Tyrosine Kinase Domain of the IR β-Subunit

Modulators in this group can be further classified into two subgroups, namely, activators or sensitizers of IR tyrosine kinase and inhibitors of the IR tyrosine kinase. 

To date, nine molecules have been reported as activators or sensitizers of the IR by acting on the IR tyrosine kinase (Table 3). These molecules are L-783281 (2,5-dihydroxy-6-(1-methylindol-3-yl)-3phenyl-1,4-benzoquinone), thioctic acid (α-lipoic acid), the chaetochromin derivatives (4548-G05 ([9,9′-bi-4H-Naphtho [2,3-b]pyran]-4,4′-dione,2,2′,3,3′-tetrahydri-5,5′,5,6′,8,8′-hexahydroxy-2,2′,3,3′-tetramethyl), TLK16998, TLK19780, and TLK19781, and three 5-substituted isophthalamides [201,202,203,204,205,206,207,208] (Figure 3). At present, tyrphostin (AG-1024), picropodophyllin (PPP), and nordihydroguaiaretic acid (INSM 18) are known to be inhibitors of the IR tyrosine kinase [209,210].

### 5.3. Regulators of the IR and IGF-1R

Regulators in this group consist of thiazolidinediones, pyrimidines, and others (Table 3).

Thiazolidinediones or thiazolidine-2,4-dione (TZDs) have recently emerged as potent antidiabetic agents, and several TZDs have been approved by the FDA for treating type 2 diabetes. Two TZDs, 5-benzylidenethiazolidine-2,4-dione and 5-(furan-2-ylmethylene) thiazolidine-2,4-dione, have been reported as selective IGF-1R inhibitors. Both of them can inhibit the IGF-1R kinase domain in vitro, with IC_50_ < 15.0 μM. This finding uncovered the potential of adding TZD derivatives to the portfolio of IGF-1R inhibitors [211].

The pyrimidine derivative PQIP (cis-3-[3-(4-methyl-piperazin-l-yl)-cyclobutyl]-1-(2-phenyl-quinoline-7-yl)-imidazo[1,5-α]pyrazin-8-ylamine) is an IGF-1R kinase inhibitor that potently inhibits the autophosphorylation of IGF-1R (IC_50_ = 19 nM) with 14-fold cellular selectivity relative to human IR [212]. Furthermore, linsitinib (OSI-906, 3-[8-amino-1-(2-phenylquinolin-7-yl) imidazo[1,5-a]pyrazin-3-yl]-1-methylcyclobutan-1-ol), a derivative of PQIP, is known to be an orally bioavailable, ATP-competitive, imidazopyrazine-based inhibitor [21,213,214].

Other chemicals such as BMS-536924 ((S)-4-[2-(3-chloro-phenyl)-2-hydroxy-ethylamino]-3-(4-methyl-6- morpholin-4-yl-1H-benzoim-idazol-2-yl)-1H-pyridin-2-one), BMS-554417 (3-[4-[(2Z)-2-[4-[[(2S)-2-(3-chlorophenyl)-2-hydroxyethyl] amino]-2-oxopyridin-3-ylidene]-7-methyl-1,3-dihydrobenzimidazol-5-yl]piperazin-1-yl]propanenitrile), and BMS-754807 ((2S)-1-[4-[(5-cyclopropyl-1H-pyrazol-3-yl)amino] pyrrolo-[2,1-*f*][1,2,4]triazin-2-yl]-N-(6-fluoropyridin-3-yl)-2-methylpyrrolidine-2-carboxamide) are inhibitors of the IR tyrosine kinase [215,216,217]. BMS-536924 and BMS-554417 belong to the pyridinones and play crucial roles in inhibiting IGF-1R and IR kinase activity [217].

Other heterocyclic compounds include the indazole derivative KW-2450 (2-thiophenecarboxamide, N-[5-[4-(2-hydroxyacetyl)-1-piperazinyl]methyl]-2-[(1E)-2-(1H-indazol-3-yl)ethenyl]phenyl]-3-methyl-4-methylbenzenesulfonate), the pyrazol derivative XL228 (4-N-(5-cyclopropyl-1H-pyrazol-3-yl)-6-(4-methylpiperazin-1-yl)-2-N-[(3-propan-2-yl-1,2-oxazol-5-yl)methyl]pyrimidine-2,4-diamine), and two pyrrole-5-carboxaldehyde analogues (2-tert-butyl-4-ethyl-3-ethyl-5-formyl-1H-pyrrole- 2,4-dicarboxylate and ethyl-5-[(tert-butylamino)carbonyl]-4-ethyl-2-formyl-1H-pyrrole-3- carboxylate) [218,219,220,221].

Additionally, three other chemicals, phenobarbital (5-Ethyl-5-phenylbarbituric acid), uric acid (7,9-dihydro-1H-purine-2,6,8(3H)-trione), and 2′-*O*-methylperlatolic acid (2-*O*-M, 4-(2-hydroxy-4-methoxy-6-pentylbenzoyl)oxy-2-methoxy-6-pentylbenzoic acid) are also effective inhibitors of the IR (Table 3). Structurally, phenobarbital and uric acid consist of a trione bond and a dione bond, respectively (Figure 3). The trione chemical phenobarbital (PB) is an IR antagonist. PB and insulin crosstalk regulated glucose through the IR in HepG2 cells [222]. The trioxopurine chemical uric acid (UA) is a final product of purine metabolism [223,224,225]. 2-*O*-M is a polyphenolic compound that binds to the extracellular domain of the IR. Combination treatment with 2-*O*-M and insulin resulted in significant activation of the insulin signaling pathway in vitro [226].

## 6. Molecular Mechanisms of Modulators Targeting the IR

### 6.1. Gene Expression of the IR Changed by DNA Methylation

**Figure 5:**
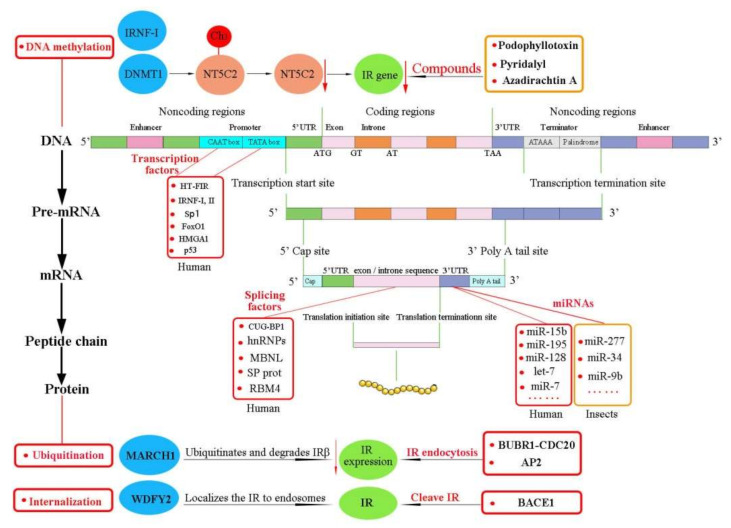
Summary of the principal IR regulators acting at the gene level (DNA methylation, xenobiotic compounds, and transcription factors at promoter), mRNA level (miRNAs at 3′UTR), and protein level (ubiquitination, endocytosis, internalization, and cleavage). DNMT1, DNA methyltransferase 1; IRNF-I, IR nuclear factor I; NT5C2, transfection 5′-nucleotidase, cytosolic II.

DNA methylation is a conserved epigenetic modification that can lead to gene silencing and regulated gene expression and the resulting cellular functions [227]. Therefore, it is indispensable for embryonic development, transcriptional regulation, and genome stability [228]. The pattern of DNA methylation in the genome is achieved by DNA methyltransferases such as DNMT1, DNMT3A, and DNMT3B [229,230,231]. DNMT1 affects IR gene expression through the transfection of 5′-nucleotidase, cytosolic II (NT5C2), an enzyme that dephosphorylates noncyclic nucleoside monophosphates into nucleoside and inorganic phosphate [232,233] (Figure 5). DNMT1 epigenetically regulates NT5C2 and the IR. Overexpression of DNMT1 stimulated DNA hypermethylation of NT5C2, leading to the silencing of the NT5C2 gene and the inhibition of IR gene expression [32,232]. Knockdown of NT5C2 induced the overexpression of DNMT1 and inhibition of the IR [232]. In contrast, overexpression of NT5C2 downregulated the expression of DNMT1 and upregulated the activation of the IR in RIN-m5F cells. In brief, NT5C2 epigenetically regulates the IR, thus potentially providing a novel therapeutic strategy. Analysis of DNA methylation might afford new diagnostic and therapeutic approaches for patients.

IR nuclear factor I (IRNF-I) also positively regulates IR promoter activity, and thus controls expression of the IR gene [32]. The methylation degree of the IRNF-I binding site within the *InR* promoter-pancreatic and duodenal homeobox 1 (PDX1) has been shown to be inversely and significantly correlated with *InR* gene expression [81]. In high-fat diet (HFD)-overfed adult offspring of the outbred Wistar rats, hypothalamic *InR* mRNA suppression was relevant to DNA hypermethylation of the *InR* promoter. Hypothalamic *InR* expression and DNA promoter methylation might lead to insulin resistance and T2DM. Interestingly, insulin resistance was more pronounced in male offspring [81]. These epigenetic phenomena were not associated with InR expression and DNA methylation alteration in female offspring, while males were predisposed. Chronic high-fat (HF) feeding decreased the insulin content and the IR level in the hippocampus [234]. New findings regarding sex-specific expression and alteration of the IR with respect to food intake and bodyweight should be considered in future studies on developmental nutritional programming.

### 6.2. Gene Expression of the IR Regulated by Xenobiotic Compounds

**Figure 6:**
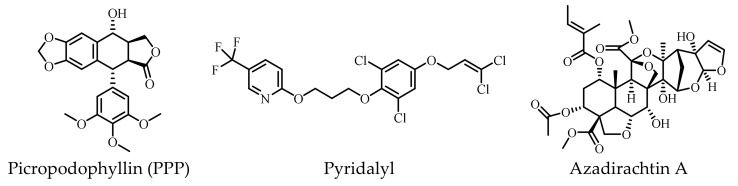
Structures of xenobiotic compounds regulating IR genes.

To date, podophyllotoxin and two commercial pesticides (pyridalyl and azadirachtin A) have been found to affect IR genes (Figure 6).

Podophyllotoxin is a plant-derived cyclolignan [235]. The medicinal and pesticidal activities of podophyllotoxin and its derivatives have recently become hot topics [236]. Podophyllotoxin has presented anti-cancer activity [237,238,239], and one of its derivatives—etoposide—is an essential anti-cancer drug included in the WHO Model List of Essential Medicines and currently in clinical use [240]. Etoposide can inhibit topoisomerase-II and promote DNA damage and apoptosis in cancer cells [241]. Interestingly, podophyllotoxin is structurally related to the IGF-1R inhibitor picropodophyllin mentioned above. Numerous studies have also reported that podophyllotoxin and its analogs possess insecticidal activity against the oriental armyworm moth *Mythimna separata* Walker, the moth *Athetis dissimilis* Hampson, and other agricultural pests [235,242,243,244,245]. RNA-seq has suggested that the podophyllotoxin derivative Compound 2a (5R,5aR,8aR,9R)-8-oxo-9-(3,4,5-trimethoxyphenyl)-5,5a,6,8,8a,9–hexahydrofuro [3’,4’:6,7] naphtha [2,3-d] [1,3] dioxol-5-yl 3-nitrobenzoate might target the IR and markedly repress the wing-development-related genes of the IR in the oriental armyworm moth [29,246]. These findings emphasize the importance of podophyllotoxin and its derivatives and provide possible guidance for further design and structural modification for the development of novel drugs and insecticidal agents acting through regulation of the IR (Figure 5).

Pyridalyl is an insecticide that has shown significant toxicity against several lepidopterous and thysanopterous pests on cotton and vegetables [247]. Oral exposure to pyridalyl resulted in upregulation of the IR (Figure 5), cytochrome P450, antioxidant enzymes, and other enzymes involved in cell death in the olive fruit fly *Bactrocera oleae* Rossi (Diptera) [248]. Activities of some enzymatic and nonenzymatic components were also changed after oral exposure to pyridalyl. Biochemical experiments have found that adult flies fed a pyridalyl-added protein hydrolysate diet presented significant mortality, with an LC_50_ value of 0.517 μg/mL. The fecundity of treated females showed no significant differences after 7 days, while the mortality of the laid eggs was obviously higher than those of controls. Activation of the IR, cytochrome P450, cell death (via apoptosis), and oxidative stress were the main alterations observed in pyridalyl-treated olive fruit flies [249]. These results are also consistent with earlier findings [250]. In a cultured cell line (*Spodoptera litura* (Fabricius), SL-1 cell), azadirachtin A, a well-known botanical insecticide, also enhanced the IR expression level. Azadirachtin A blocked the combination of insulin and IR, leading to a change of phosphorylation of the InR, thus affecting phosphorylation in the downstream signaling pathway [251].

### 6.3. IR Expression Regulated Posttranscriptionally via MicroRNAs

MicroRNAs (miRNAs) are evolutionarily conserved small noncoding RNAs that can inhibit translation or promote mRNA degradation [252].

A growing number of studies have shown that miRNAs are critical components of posttranscriptional gene expression regulation and are critical in biological processes for humans and insects [253,254,255]. Different miRNAs have been reported to participate in human IR modulation [11,57] (Figure 4); for example, Let-7 miRNA family members regulated the IR expression in human HEK293T cells and pancreatic ductal adenocarcinoma (PDAC) [256,257]. Let-7 family members also targeted and downregulated the IR/IGF pathway in PDAC; miR-7 is a brain-abundant miRNA that targets the IR, IRS-2, and insulin-degrading enzyme (IDE), and thus affects insulin signaling through posttranscriptional regulation of the IRS-2, IR, and IDE pathways in AD patients [258]. Consequently, miRNAs might thus provide an effective target for the development of IR/IGF pathway-specific treatment strategies.

Further, miRNAs are known to regulate development, metabolism, reproduction, and many physiological syntheses in insects. In mosquitoes, miR-277 targets ILP7 and ILP8 and acts as a monitor to control ILP7 and ILP8 mRNA levels [259]. Additionally, miRNAs have functions during wing development [260]; miR-34 mediates the crosstalk between JH, 20E, and IIS pathways by targeting two binding sites in the 3′UTR of *NlIR1,* and formed a positive autoregulatory loop to control wing morphs in *N. lugens* [120]. Another study reported that miR-9b played a key role in regulating wing dimorphism in the brown citrus aphid *Aphis citricidus* (Kirkaldy) [260]. Likewise, miR-9b-ABCG4-insulin signaling has been shown to be involved in the transition from the fourth instar winged nymph to the winged adult. Inhibition of *Aci-miR-9b* increased the proportion of winged offspring under normal conditions, while overexpression of aci-miR-9b resulted in a decline in the proportion of winged offspring under crowded conditions and resulted in malformed wings in adults [260]. These results enrich our knowledge of miRNA as an important factor that could help insects adapt to changing environments. Such information might stimulate the development of methods for controlling migratory insect pests and the corresponding viral diseases transmitted by these insects.

### 6.4. Regulation of Expression of the IR Protein

#### 6.4.1. Ubiquitination of the IR

Dynamic modulation and posttranslational modification of proteins are multistep enzymatic biological processes that occur in response to physiological cues [261]. Ubiquitination is one of the most common forms of posttranslational dynamic modification of proteins in cells [262]. Thousands of proteins are targeted for ubiquitination at some time during their life. Ubiquitination regulates extensive complex physiological processes, including protein degradation and interactions, endocytosis, and cell-cycle progression and differentiation, as well as the activation or inactivation of substrates [261]. Dysregulation of ubiquitination contributes to various diseases [263], and both IR and IRS proteins are regulated by ubiquitination [264,265,266,267] (Figure 5).

**Figure 7:**
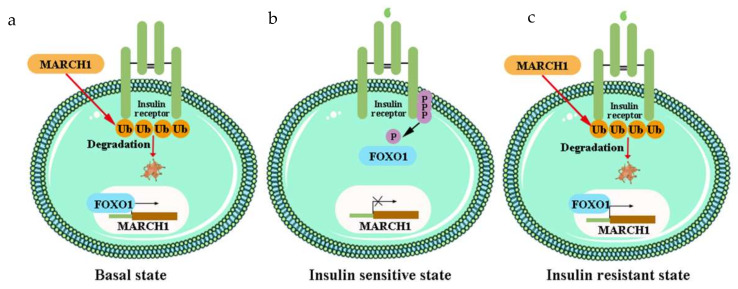
MARCH 1 regulates the IR: (**a**) MARCH 1 ubiquitinates and degrades the β-subunit of the IR, thereby decreasing IR surface expression; (**b**) IR activation inhibits FOXO, resulting in transcriptional repression of MARCH 1 and an increase in surface IR levels; (**c**) Insulin fails to inhibit FOXO, leading to enhanced MARCH 1 expression, reduced surface IR levels, and an impaired IIS pathway. Ub, ubiquitin. FOXO, forkhead transcription factor subgroup O.

The negative regulator E3 ubiquitin ligase MARCH 1 impairs cellular insulin action by degrading the cell surface IR. MARCH 1 ubiquitinates the IR to decrease the level of cell surface IR in the basal state, rather than after insulin stimulation [267]. This is different to other IR ubiquitin ligases. MARCH 1 also controls IR tyrosine phosphorylation, regulation of PTP1B, variation in clathrin-mediated IR endocytosis, and alteration of IR compartmentation into lipid rafts and caveolae [267]. MARCH 1 is conducive to the pathophysiology of T2DM, and thus could provide a novel therapeutic approach (Figure 7) [32,267].

#### 6.4.2. Endocytosis of the IR

**Figure 8:**
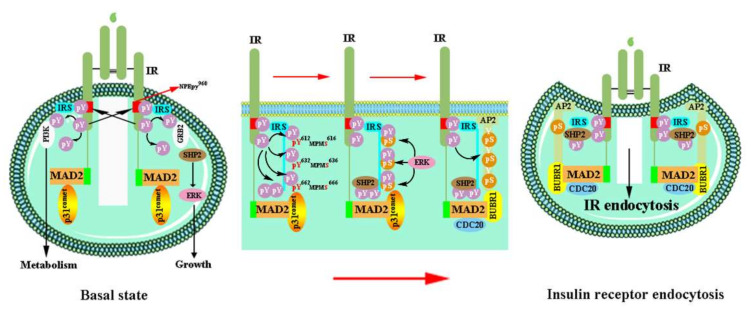
Activated IR endocytosis, regulated by the IRS and MAD2. The IRS proteins directly bind to the IR-JM domain and interact with AP2; p31^comet^ suppresses the interaction of BUBR1-CDC20-AP2 and IR-bound MAD2, thus interfering with IR endocytosis in the basal state. The connection of BUBR1-CDC20-AP2 and IR-MAD2 causes IR endocytosis. SHP2, Src homology phosphatase 2; ERK, extracellular-signal-regulated kinase; AP2, assembly polypeptide 2; Y612/Y632/Y662, IR tyrosine phosphorylates the YXXΦ motifs on the IRS1. S616/S636/S666, activate ERK phosphorylates on the IRS1.

Protein ubiquitination acts as a signal for sorting, trafficking, and membrane protein removal by endocytosis [261]. Active IR is internalized by dynamin-mediated endocytosis [268]. Therefore, IR endocytosis is a crucial factor that regulates the insulin signaling intensity and duration (Figure 5). The IRS module and MAD2 protein collaborate to trigger and regulate activated IR endocytosis [56] (Figure 8). The IRS module is activated by the SHP2-MAPK pathway [56,269]. The phosphotyrosine-binding domain of the IRS is directly bound to the phosphorylated NPEpy^960^ motif in the IR-JM domain, interacts with assembly polypeptide 2 (AP2), and then triggers IR endocytosis. Src homology phosphatase 2 (SHP2) binds to the C-terminal phosphotyrosine sites on the IRS1 and dephosphorylates pY612/pY632/pY662 of the doubly phosphorylated IRS1 (pY/pS) to facilitate the IRS1–AP2 interaction.

SHP2 promotes IR endocytosis directly by removing IRS tyrosine phosphorylation and indirectly by activating the MAPK pathway [56]. In the MAD2-dependent module, MAD2 binds to the C-terminal MAD2-interacting motifs (MIMs) of IR then interacts with BUBR1-CDC20 and provides another binding site for AP2 [270]. The successful connection of BUBR1-CDC20-AP2 and IR-MAD2 leads to IR endocytosis in cells. In the basal state, p31^comet^ disrupts IR endocytosis by inhibiting the interaction between BUBR1-CDC20-AP2 and the IR-bound MAD2 [271]. Persistent hyperinsulinemia also accelerates IR endocytosis and impairs the functional IR level at the PM. SHP2 inhibition provides a hopeful means to interfere with the endocytosis feedback loop, prolonged insulin signaling at the PM, and potentiated insulin sensitivity [271]. These findings support that the feedback regulation of IR endocytosis may contribute to diabetes therapy in human patients.

#### 6.4.3. Endosome Localization and Cleavage of the IR

**Figure 9:**
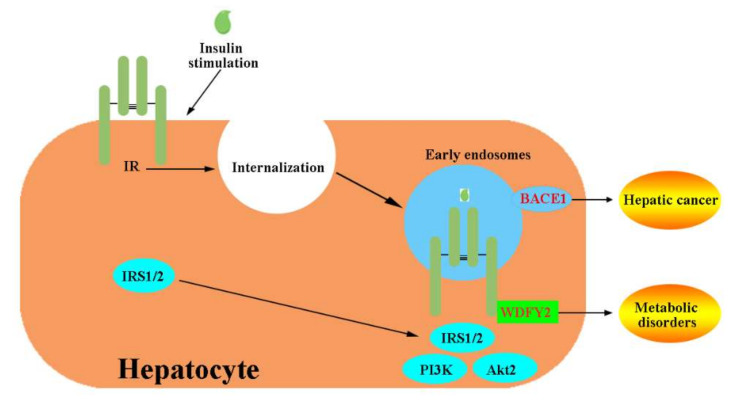
Model for the role of WDFY2 and BACE1 in IR internalization into endosomes in hepatocytes. After insulin stimulation, the IR internalizes into endosomes. WDFY2 interacts with the IR to localize it to endosomes such that downstream IRS1/2 and AKT2 can be recruited to the endosomal IR. BACE1, located at the early endosomes, cleaves the IR-ECD and decreases the number of mature IRs. WDFY2, WD Repeat and FYVE domain containing 2; BACE1, β-site amyloid precursor protein cleaving enzyme 1.

The liver is the largest parenchymal organ, regulating detoxification in the body and playing a fundamental role in coordinating systemic metabolic homeostasis [15,272]. In hepatocytes, IR ligand binding results in IR-TK autophosphorylation and internalization to generate intracellular signaling endosomes, which plays an important role in activating the hepatic insulin-evoked PI3K-AKT pathway [273] (Figure 5). The IR continues to signal at endosomal compartment Akt. Akt isoform 2 (Akt2), the most abundant Akt isoform in tissues, is also recruited to the endosomes and exhibits higher specific enzymatic activity than PM. The endosome-located protein WD Repeat and FYVE domain containing 2 (WDFY2) is highly expressed in the liver [274,275] and has been reported to act as an adaptor-like protein for the regulation of protein phosphorylation and endocytosis [276]. WDFY2 interacts with the IR through its WD1-4 domain and localizes the IR to endosomes after insulin stimulation, ensuring that downstream IRS1/2 is recruited [275] (Figure 9). Disturbing the IR-WDFY2 interaction ultimately impairs downstream PI3K-AKT signaling. Therefore, increasing WDFY2 liver expression might provide a new treatment concept for metabolic disorders.

In the liver, β-site amyloid precursor protein cleaving enzyme 1 (BACE1), a therapeutic target of AD [277], regulates the number of IRs and insulin signaling in a glucose concentration-dependent manner [21]. BACE1 is located in the trans-Golgi network (TGN), PM, and early endosomes [278] (Figure 9). BACE1 can cleave IR-ECD and decrease the number of mature IRs [21]. BACE1 inhibition recovered functional IR and enhanced insulin signaling in diabetes patients. A soluble truncated IR (IRsol, especially the IR α-subunit) has been shown to be elevated in the plasma of T2DM patients compared to control groups [279,280]. Consequently, the use of BACE1 inhibitors may potentially assist in hepatic cancer management, and the cleaved IR might serve as a novel biomarker for hepatic cancer diagnosis and management.

## 7. Conclusions and Future Perspectives

**Figure 10:**
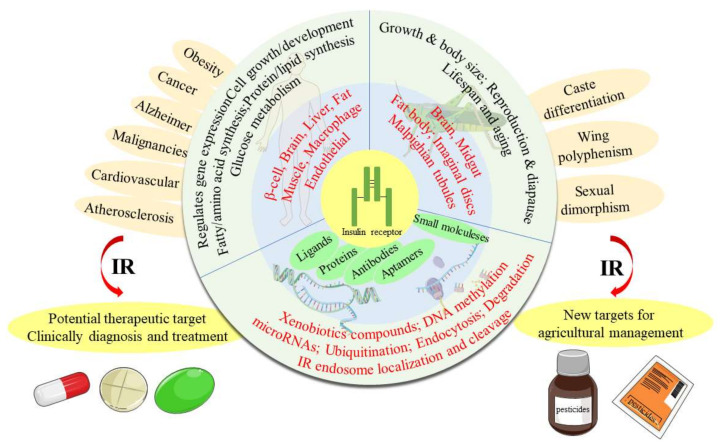
Summary of the contents and the conclusions.

Briefly, the contents and the conclusions of this paper are summarized in the following diagram (Figure 10).

Since the discovery of the IR, the structure and signaling pathways of this vital receptor have been systematically studied. Structurally, the IR-ECD converts the overall architecture from an autoinhibited inverted “V” shape into a “T”-shaped conformation after insulin binding, where at least one insulin molecule at two sites and a maximum of four insulin molecules at four sites are required to form the “T”-shaped dimer. Strikingly, insulin binds to the majority of IRs in a symmetric manner.

Functionally, the IR plays essential roles in metabolism, cell growth, development, and numerous physiological processes. Moreover, the IR has been related to the most prevalent diseases, such as T2DM and cancers, and thus has been considered as a novel therapeutic target of IR-related diseases in humans [263]. In-depth analysis of IR regulators, including insulin, insulin analogs, insulin-mimetic peptides, antibodies, aptamers, and proteins, would help develop an understanding of the regulation of cellular IIS pathways, substantially contributing to the development of novel drugs for T2DM and other diseases related to insulin signaling [281]. Therefore, design and synthesis of novel small molecules as oral drugs has been regarded as a source of potential IR activation modulators. Metformin is a good example that has emerged in recent years.

Noteworthy, there exists high conservation in the IIS pathway between mammals and insects [5]. This has led to the possibility for the IR to be used as a potential target for the development of new insecticides in order to control insects. To date, insecticides have been claimed as exactly targeting the IR, even though the IR is known to play roles in caste differentiation, wing polyphenism, and some other important physiological processes. Moreover, IR-silenced insects are blocked at the larval–pupal transformation and then die [107]. It is notable that podophyllotoxin, pyridalyl, and azadirachtin A affect the IR.

In summary, these discoveries are expected to be useful for the development of novel oral drugs and insecticidal agents targeting the IR. DNA methylation, microRNAs, protein ubiquitination and endocytosis, endosome localization, and cleavage are remarkable categories of IR regulation to which significant attention should be paid in the development of novel drugs and insecticides.

## Figures and Tables

**Table 1:** Summary of the available structures of IR.

Classification	Structure of IR	References
Domain layout	an (αβ)2 disulfide-linked homodimer	[35]
cDNA sequenced	α chain lies on the N-terminal of the β chain	
3D structure of human apo IR ectodomain	intracellular unphosphorylated from TKD (2.1 Å resolution, PDB 1IRK)	
	receptor’s isolated L1-CR-L2 module (2.32 Å resolution, PDB 2HR7)	
	intact receptor ectodomain in apo form (3.8 Å resolution, PDB 2DTG)	
CryoEM structures of IR	insulin holoreceptor (full-length receptor inclusive of transmembrane and cytoplasmic elements)	[42]
	isolated receptor ectodomain	[41,43]
	an ectodomain construct (leucine-zippered receptor ectodomain)	[44]

**Table 3:** Small-molecule positive modulators of the IR.

Group	Compound	Pharmacological Activity	Experimental Model	References
1	Thymolphthalein (TP) weak agonist	displaces insulin from IR, binds to the IR; ↑auto- and substrate-based phosphorylation of IR	isolated primary mouse adipocytes	[183]
1	Dicholine succinate (DS) (sensitizers)	↑IR-mediated signaling	mice	[184,185]
1	GW501516	↑expression of the IR (1.3-fold than insulin); ↓TNF-α (tumor necrosis factor α)-induced IR expression	differentiated 3T3-L1 adipocytes	[186]
1	DDN (activator)	↑phosphorylation of Akt and ERK (bind to IR-TKD); ↓blood glucose	male C57BL/6J, C57BL/KsJ *db/db* mice; female C57BL/KsJ *ob/ob* mice	[187]
1	CSN (activator)	↑IR phosphorylation (time-dependent manner)		
1	Ceritinib (LDK378) (off-target inhibitor)	↓IGF-1R (IC_50_: 8 nm) phosphorylation and downstream effector AKT; ↓IR (IC_50_: 7 nm) phosphorylation	human primary cell culture PhKh1 of a pediatric HGNET-BCOR patient (P1)	[188,189,190]
1	Penta-*O*-galloyl-D-glucose (PGG)	↑phosphorylation of the IR and Akt (α-PGG/β-PGG isoform)	3T3-L1 adipocytes	[191,192]
1	6Cl-TGQ	↑IR (without activating IGF-1R); ↑glucose uptake	3T3-L1 adipocytes	[193]
1	Adenosine	↑phosphorylation and activation of IR (interacted with IR-α)	HepG2 liver cells; insulin-resistant T2D Leprdb/db mice	[194]
1	Gingerenone A (Gin A)	↑tyrosine phosphorylation of the IR; ↑translocation of GLUT4; ↑insulin-stimulated glucose uptake	murine 3T3-L1 adipocytes; rat L6 myotubes	[195]
1	Ursolic acid	↑autophosphorylation of the β-subunit of the IR; ↑glucose uptake (dose-dependent manner)	3T3-L1 adipocytes	[196,197]
1	Metformin	↑autophosphorylation of the human IR (activator of AMP-activated protein kinase (AMPK)) lactic acidosis	CHO cells expressing the human IR	[198,199]
1	Rutaecarpine (activator)	↑autophosphorylation of the human IR (bind to IR-ECD)		[200]
2	L-783,281 (insulin mimetic) (activator)	↑phosphorylation of the IR β subunit & IRS-1; ↑PI 3-kinase activity (13); ↑phosphorylation Akt kinase; ↑glucose uptake	Chinese hamster ovary cells (overexpress the human IR) (CHO.IR)	[201]
2	Thioctic acid (α-lipoic acid) (activator)	↑activation of the IR (bind to IR-TKD)	mice primary hepatocytes	[202]
2	4548-G05 (insulin mimetics) (activator)	↑phosphorylations of IR, IRS-1, Akt	C2C12 myotubes [197]	[203]
2	TLK16998 (sensitizer)	↑IR autophosphorylation (activates IR-TKD β-subunit); ↑IRS-1 phosphorylation;↑PI3-kinase recruitment;↑GLUT4 translocation; ↑glucose uptake	3T3-L1 adipocytes	[204]
2	TLK19780 (activator)	↑the amount of autophosphorylated IR;	HTC-IR cells	[205]
2	TLK19781	↑phosphorylation of the IR-TKD; ↑GLUT4 translocation;↑glucose transport	3T3-L1 fibroblasts	[206,207]
2	5-substituted isophthalamides (sensitizer)	IR sensitizer, inactive without insulin	3T3-L1 adipocytes	[208]
2	Tyrphostin (AG-1024)	↓autophosphorylation IGF-1R (IC_50_:0.4 μM)/IR (IC_50_:0.1 μM)	NIH-3T3 fibroblasts	[20]
2	Picropodophyllin (PPP)	↓IGF-1R autophosphorylation at the substrate level	mice	[209]
2	Nordihydroguaiaretic acid (INSM-18)	↓activation of the IGF-1R (inhibitor); ↓phosphorylation of the Akt/PKB serine kinase	MCF-7 human breast cancer cells	[210]
3	5-benzylidenethiazolidine-2,4-dione	↓IGF-IR and IR kinase activity	MCF-7 human breast cancer cell line	[211]
3	5-(furan-2-ylmethylene) thiazolidine-2,4-dione	↓IGF-IR and IR kinase activity		
3	PQIP	↓autophosphorylation of the IGF-1R (IC_50:_ 19 nmol/L)	3T3/huIGF1R fibrosarcoma cells	[212]
3	linsitinib (OSI-906)	↓IR/IGF-1R kinase; ↓autophosphorylation of IR and IGF-1R (human)	3T3/huIGF-1R fibrosarcoma cells; GEO human colorectal cancer cells	[213]
3	Compound 9b	↓phosphorylation of IR and IGF-1R; ↓pAkt	GEO human colorectal tumor cell line/tumor xenograft models	[214]
3	BMS-536924	↓phosphorylation of the IR and IGF-1R tyrosine kinase; ↓Akt and MAPK phosphorylation	Sal tumor model	[215]
3	BMS-554417	↓IGF-IR and IR kinase activity and proliferation	carcinoma cell lines (Colo205 and OV202)	[216]
3	BMS-754807	↓phosphorylation of IGF-1R and IR	postmenopausal, estrogen-dependent breast cancer	[217]
3	KW-2450	↓tyrosine kinase of IR (IC_50:_ 5.64 nM)/IGF-1R (IC_50:_ 7.39 nM)	HT-29/GFP colon cancer xenograft model	[218,219]
3	XL228	↓tyrosine kinase of IGF-1R and other protein kinases	patients with advanced malignancies	[220]
3	pyrrole-5-carboxaldehyde analogues	↓tyrosine kinase of IR and IGF-1R; ↓autophosphorylation of IR and IGF-1R	human embryonic kidney cells (HEK-293)	[221]
3	Phenobarbital (PB) (antagonist)	↓dephosphorylate-activated IR; ↓dephosphorylation of phosphorylated Akt/FOXO1	primary hepatocytes and HepG2 cells; mouse	[222]
3	Uric acid (UA)	induced ENPP1 binding to IR α-subunit; ↓autophosphorylation of IR and IRS; ↓glucose transport	human umbilical vein endothelial cell	[223,224,225]
3	2′-O-methylperlatolic acid (sensitizer)	↑insulin signaling pathway (binds to IR-ECD); ↑cellular glucose uptake	Hepa and C2C12 myotubes	[226]

GLUT4, glucose transporter 4; HGNET-BCOR, high-grade neuroepithelial tumor with BCOR alteration; ENPP1, ectonucleotide pyrophosphatase/phosphodiesterase 1.
